# Supplementary material for: Cognitive impairment at older ages among 8000 men and women living in Mexico City: a cross-sectional analyses of a prospective study
Source: BMC Public Health. 2024 Dec 31;24:3620. doi: 10.1186/s12889-024-21093-5 (PMC11687187; doi:10.1186/s12889-024-21093-5)
Supplement: Supplementary file 1 — Supplementary Material 1 [file 12889_2024_21093_MOESM1_ESM.docx]

**Supplementary Material**

**Cognitive impairment at older ages among 8000 men and women living in Mexico City: cross-sectional analyses of a prospective study**

**Supplemental Tables**

| 1. Age- and sex-specific mean MMSE scores and prevalences of cognitive impairment, **by district** | 2 |
| --- | --- |
| 1. Age- and sex-specific mean MMSE score and prevalence of cognitive impairment **including those who reported having no formal education** | 3 |

**Supplemental Figures**

| 1. Selection of participants | 4 |
| --- | --- |
| 1. Prevalence of cognitive impairment and mean MMSE **including those who reported having no formal education** | 5 |
| 1. Sex-specific distribution of MMSE scores who reported having had at least some level of formal education | 6 |
| 1. District-standardised sex-specific prevalences of cognitive impairment with and without key metabolic risk factors, by age and sex | 7 |

| **Supplemental Table 1.** **Age- and sex-specific mean MMSE scores and prevalences of cognitive impairment, by district** | | | | | | | | | |
| --- | --- | --- | --- | --- | --- | --- | --- | --- | --- |
| **Coyoacán** | | | | | | | | | |
|  | **Men** | | | **Women** | | | **Overall** | | |
| **Age group** | ***n*** | **MMSE**  **mean (SD)** | **Prevalence**  **%** | ***n*** | **MMSE**  **mean (SD)** | **Prevalence**  **%** | **N** | **MMSE**  **mean (SD)** | **Prevalence**  **%** |
| 50-59 | 353 | 27.2 (2.5) | 11 | 807 | 27.2 (2.7) | 14 | 1160 | 27.2 (2.6) | 12 |
| 60-69 | 457 | 26.7 (3) | 19 | 1116 | 26.4 (3) | 22 | 1573 | 26.6 (3) | 20 |
| 70-79 | 426 | 25.3 (4) | 34 | 654 | 24.8 (3.7) | 38 | 1080 | 25.1 (3.8) | 36 |
| 80-89 | 181 | 22.9 (4.8) | 54 | 254 | 22.3 (4.8) | 63 | 435 | 22.6 (4.8) | 59 |
| **All** | **1417** | **25.5 (3.6)** | **30** | **2831** | **25.2 (3.6)** | **34** | **4248** | **25.4 (3.6)** | **32** |
| **Iztapalapa** | | | | | | | | | |
|  | **Men** | | | **Women** | | | **Overall** | | |
| **Age group** | ***n*** | **MMSE**  **mean (SD)** | **Prevalence**  **%** | ***n*** | **MMSE**  **mean (SD)** | **Prevalence**  **%** | **N** | **MMSE**  **mean (SD)** | **Prevalence**  **%** |
| 50-59 | 342 | 28 (1.9) | 6 | 1021 | 27.9 (2.3) | 10 | 1363 | 27.9 (2.1) | 8 |
| 60-69 | 347 | 27.3 (2.9) | 12 | 874 | 27 (2.8) | 17 | 1221 | 27.2 (2.8) | 14 |
| 70-79 | 298 | 25.8 (3.7) | 26 | 632 | 25.5 (3.8) | 31 | 930 | 25.6 (3.7) | 28 |
| 80-89 | 161 | 23.7 (4.3) | 49 | 274 | 23 (5.1) | 54 | 435 | 23.3 (4.7) | 52 |
| **All** | **1148** | **26.2 (3.2)** | **23** | **2801** | **25.8 (3.5)** | **28** | **3949** | **26 (3.3)** | **26** |
| MMSE, Mini-Mental State Examination. Cognitive impairment defined as MMSE ≤24. Estimates shown among 8,197 participants aged 50 to 89 years at resurvey who reported having had some level of education. Overall prevalences are the simple averages of the age and sex-specific estimates (i.e., are uniformly standardised). | | | | | | | | | |

| **Supplemental Table 2. Age- and sex-specific mean MMSE score and prevalence of cognitive impairment including those who reported having no formal education** | | | | | | | | | |
| --- | --- | --- | --- | --- | --- | --- | --- | --- | --- |
|  | **Men** | | | **Women** | | | **Overall** | | |
| **Age group** | ***n*** | **MMSE**  **mean (SD)** | **Prevalence**  **%** | ***n*** | **MMSE**  **mean (SD)** | **Prevalence**  **%** | **N** | **MMSE**  **mean (SD)** | **Prevalence**  **%** |
| 50-59 | 710 | 27.4 (2.8) | 10 | 1913 | 27.2 (3.1) | 15 | 2623 | 27.3 (2.9) | 13 |
| 60-69 | 846 | 26.6 (3.5) | 19 | 2205 | 26 (3.7) | 26 | 3051 | 26.3 (3.6) | 22 |
| 70-79 | 806 | 25 (4.2) | 35 | 1629 | 23.9 (4.6) | 46 | 2435 | 24.4 (4.4) | 40 |
| 80-89 | 426 | 22.5 (4.8) | 58 | 753 | 20.9 (5.7) | 68 | 1179 | 21.7 (5.3) | 63 |
| **All** | **2788** | **25.4 (3.8)** | **31** | **6500** | **24.5 (4.3)** | **39** | **9288** | **24.9 (4.1)** | **35** |
| MMSE, Mini-Mental State Examination. Cognitive impairment defined as MMSE ≤24. Estimates shown among 9,288 participants aged 50 to 89 years at resurvey who reported having had some level of education. Overall prevalences are the simple averages of the age and sex-specific estimates (i.e., are uniformly standardised). | | | | | | | | | |

**Supplemental Figure 1. Selection of participants**

**
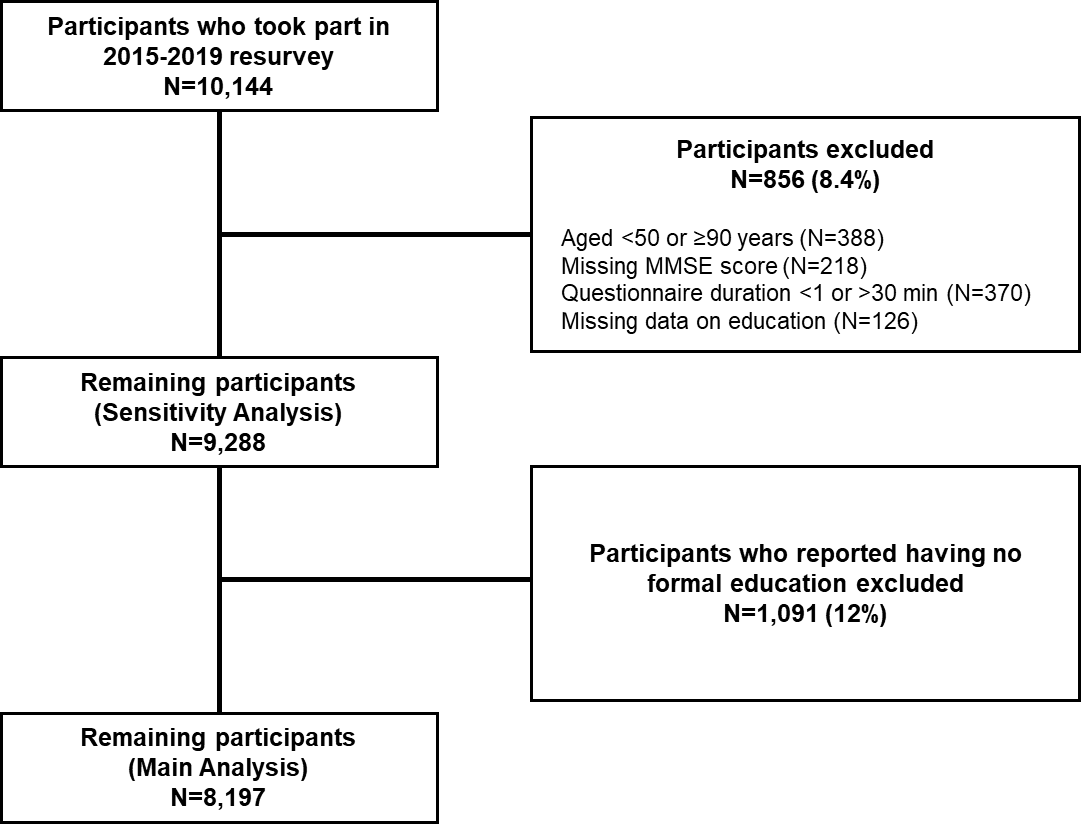
**

| **Supplemental Figure 2. Prevalence of cognitive impairment and mean MMSE including those who reported having no formal education** |
| --- |
| **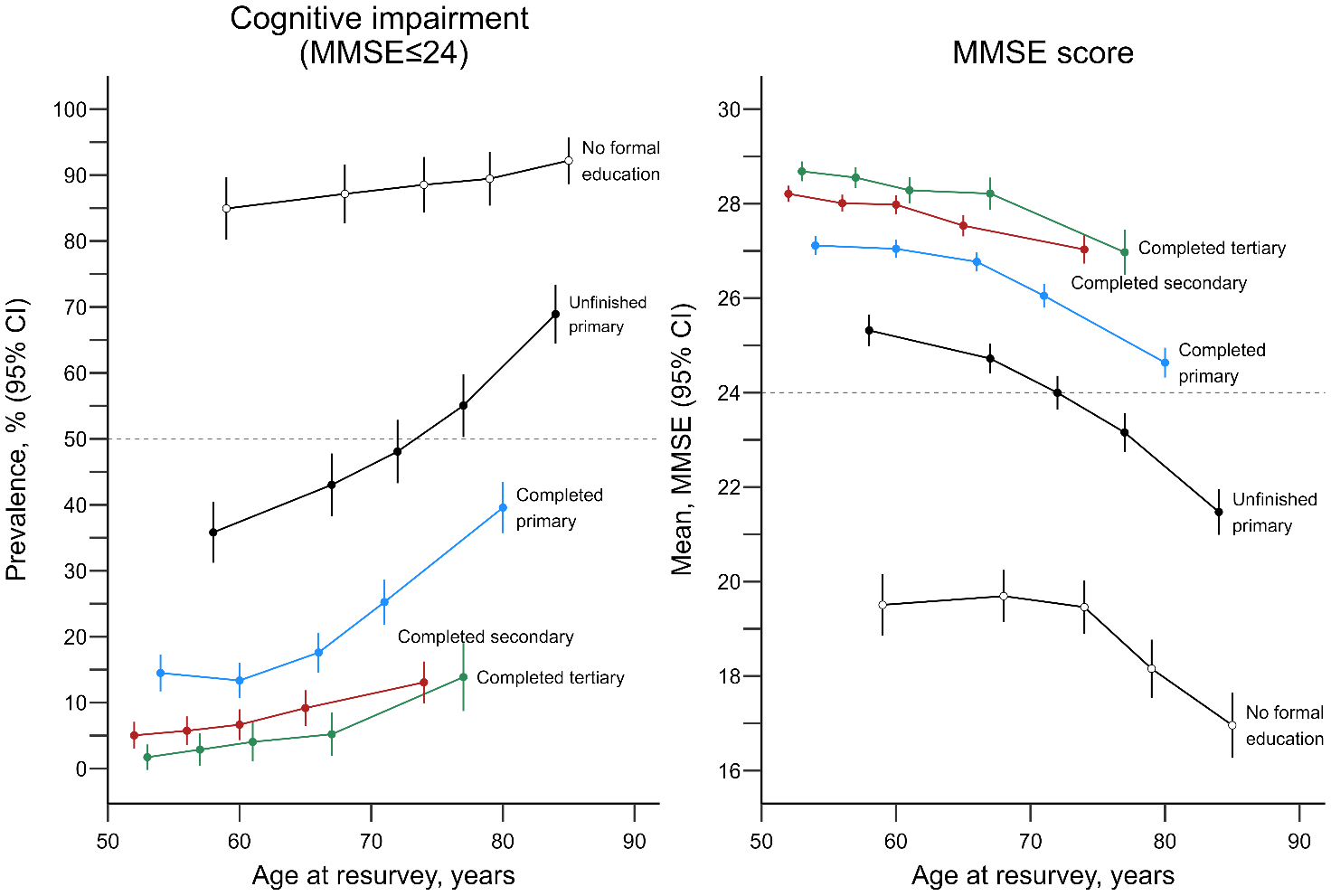** |
| MMSE, Mini-Mental State Examination; CI, confidence interval. Estimates shown among 9,288 participants aged 50 to 89 years at the 2015-2019 resurvey. Unadjusted prevalences and means with 95% CIs are shown. Each point involves ~210 participants with no formal education, ~410 with unfinished primary, ~610 with completed primary, ~430 with completed secondary, and ~170 with completed tertiary education. |

| **Supplemental Figure 3. Sex-specific distribution of MMSE scores who reported having had at least some level of formal education** |
| --- |
| **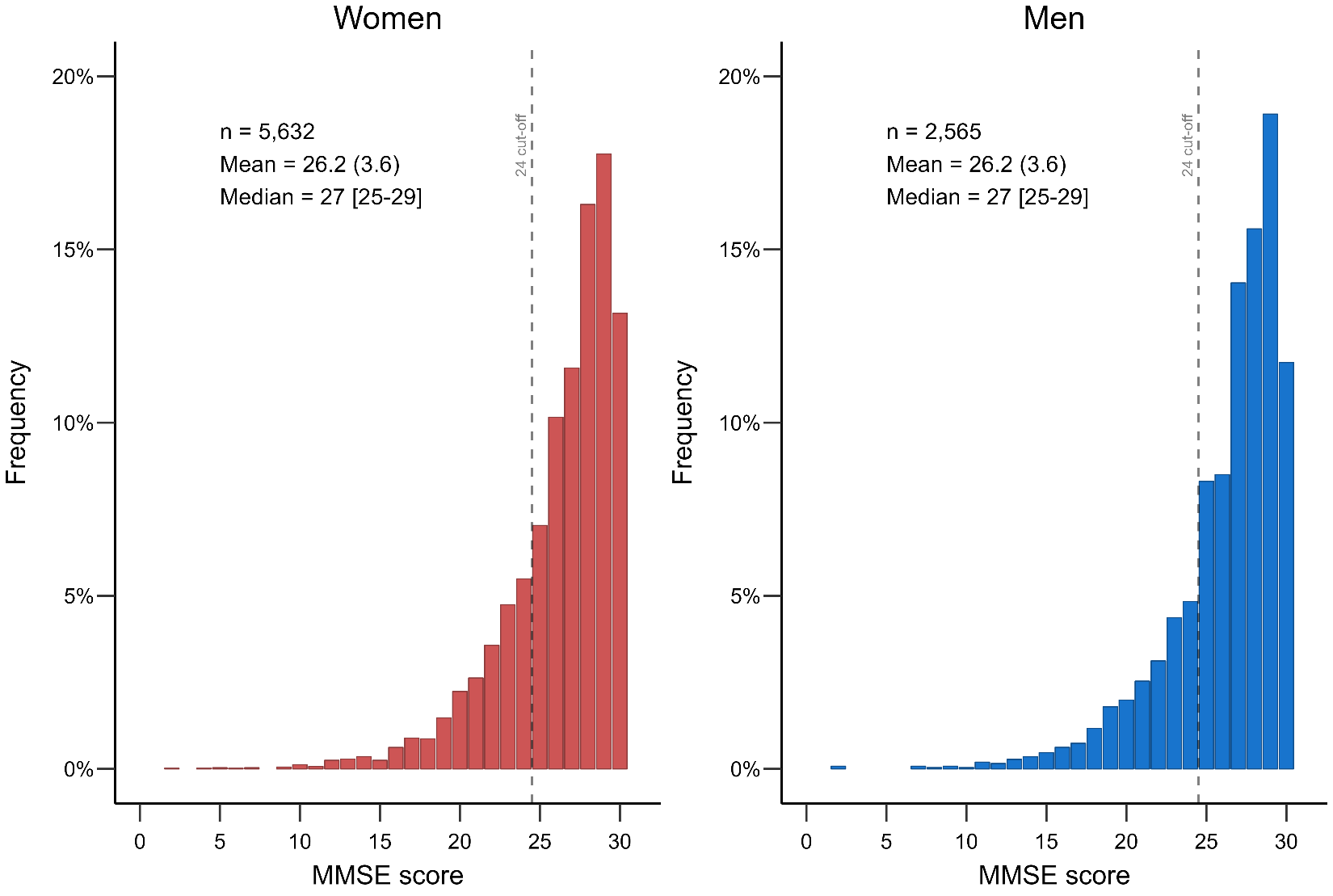** |
| MMSE, Mini-Mental State Examination. Exclusion criteria listed in Supplementary Figure 1. Data shown among 8,197 participants aged 50 to 89 years at the 2015-2019 resurvey. |

| **Supplemental Figure 4. District-standardised sex-specific prevalences of cognitive impairment with and without key metabolic risk factors, by age and sex** | |
| --- | --- |
| **Women** | **Men** |
| **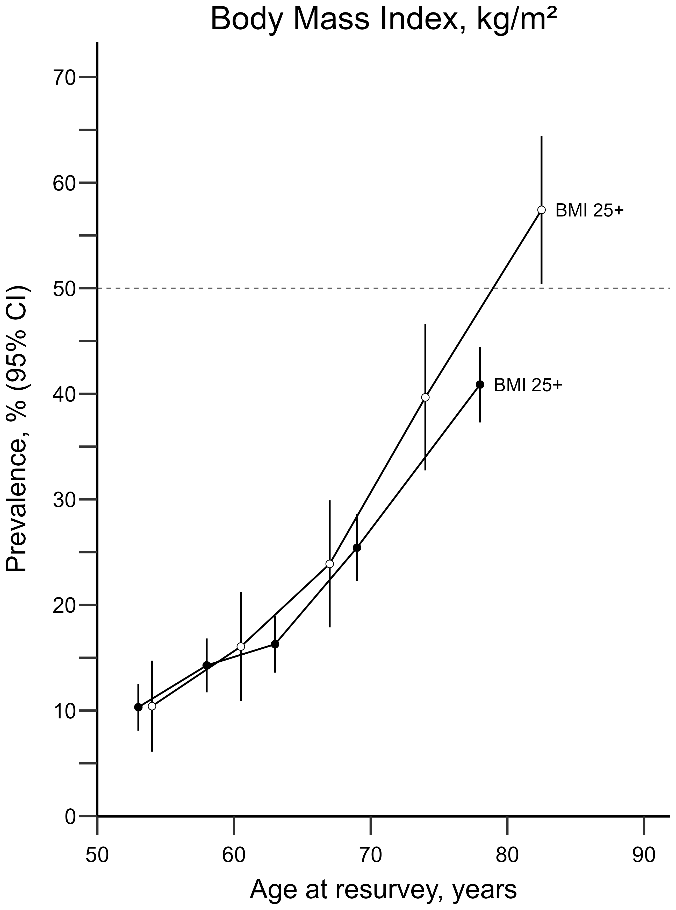** | 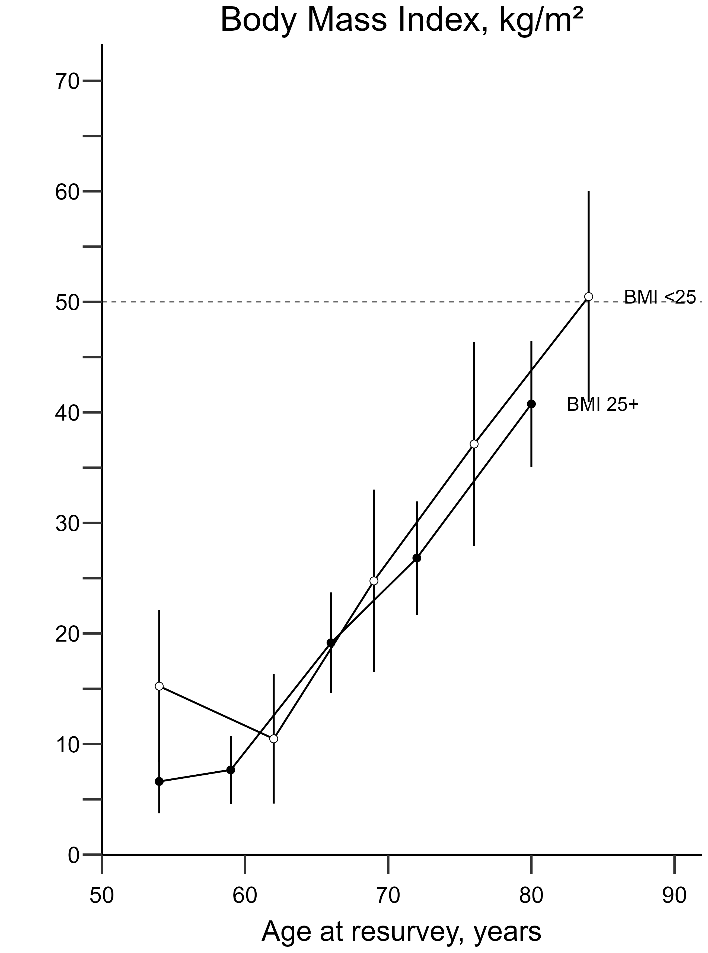 |
| **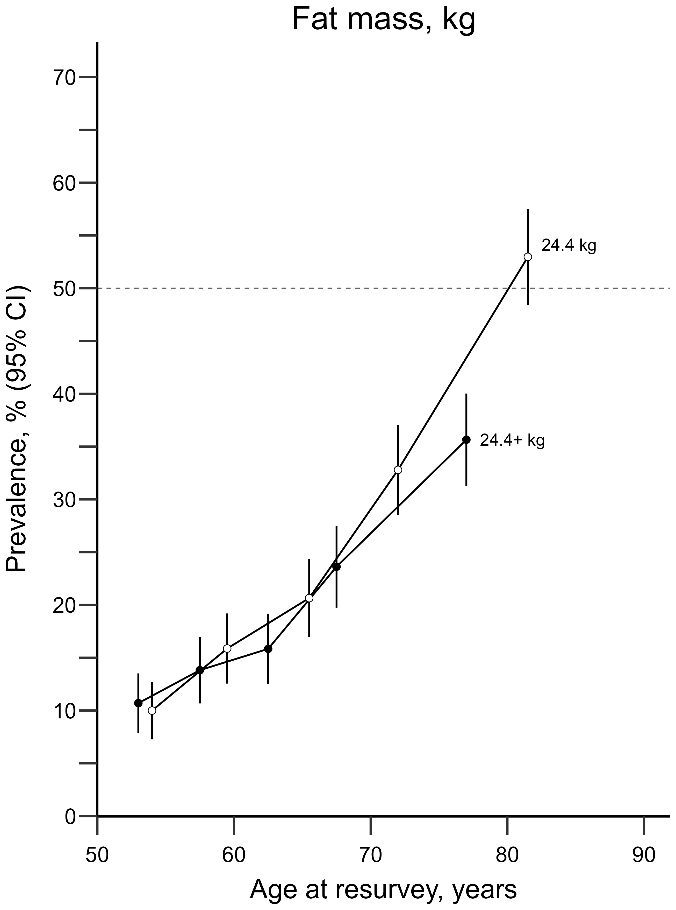** | **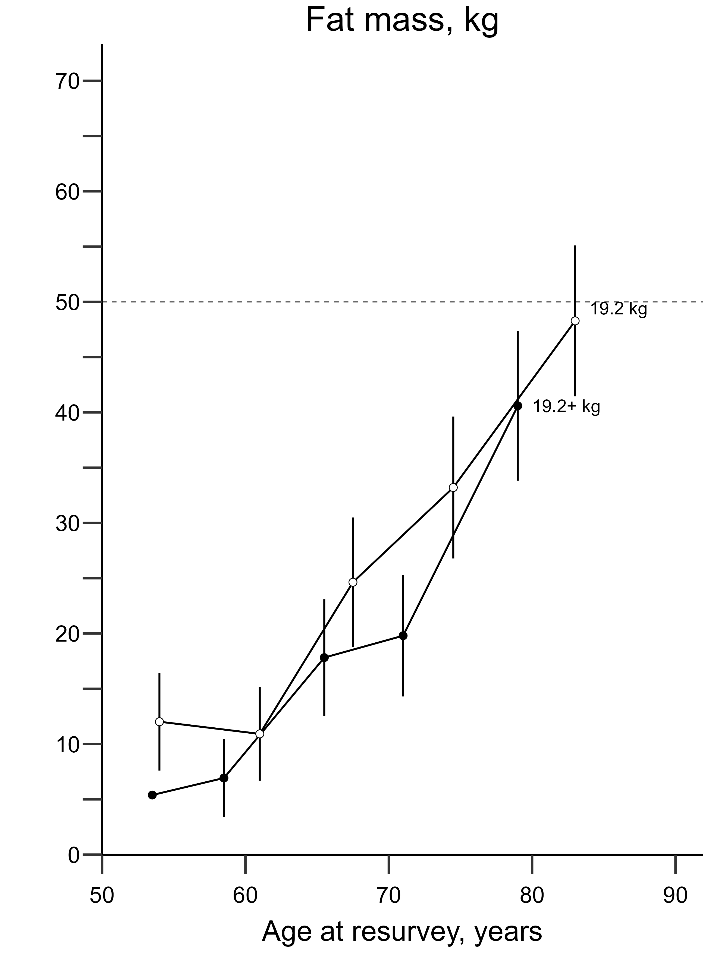** |
| MMSE, Mini-Mental State Examination; CI, confidence interval. Sex-specific median fat mass (19.2 kg for men and 24.4 kg for women) were used as cut-offs. Estimates shown among participants aged 50 to 89 years at the 2015-2019 resurvey who reported having had some level of education, for women 4,567 participants for the left-top panel and 4,627 the bottom-left panel. For men, 2,019 participants for the top-right panel and 2,049 for the bottom-right panel. | |
